# Application of Design Structure Matrix to Simulate Surgical Procedures and Predict Surgery Duration

**DOI:** 10.1155/2021/6340754

**Published:** 2021-12-06

**Authors:** Zhaoxuan Li, Derrick Tate, Thomas McGill, John Griswold, Ming-Chien Chyu

**Affiliations:** ^1^Healthcare Engineering Graduate Program, Lubbock, TX, USA; ^2^Department of Mechanical Engineering, Texas Tech University, Lubbock, TX, USA; ^3^Department of Surgery, School of Medicine, Texas Tech University Health Sciences Center, Lubbock, TX, USA

## Abstract

**Background:**

The complexities of surgery require an efficient and explicit method to evaluate and standardize surgical procedures. A reliable surgical evaluation tool will be able to serve various purposes such as development of surgery training programs and improvement of surgical skills.

**Objectives:**

(a) To develop a modeling framework based on integration of dexterity analysis and design structure matrix (DSM), to be generally applicable to predict total duration of a surgical procedure, and (b) to validate the model by comparing its results with laparoscopic cholecystectomy surgery protocol.

**Method:**

A modeling framework is developed through DSM, a tool used in engineering design, systems engineering and management, to hierarchically decompose and describe relationships among individual surgical activities. Individual decomposed activities are assumed to have uncertain parameters so that a rework probability is introduced. The simulation produces a distribution of the duration of the modeled procedure. A statistical approach is then taken to evaluate surgery duration through integrated numerical parameters. The modeling framework is applied for the first time to analyze a surgery; laparoscopic cholecystectomy, a common surgical procedure, is selected for the analysis.

**Results:**

The present simulation model is validated by comparing its results of predicted surgery duration with the standard laparoscopic cholecystectomy protocols from the *Atlas of Minimally Invasive Surgery* with 2.5% error and that from the *Atlas of Pediatric Laparoscopy and Thoracoscopy* with 4% error.

**Conclusion:**

The present model, developed based on dexterity analysis and DSM, demonstrates a validated capability of predicting laparoscopic cholecystectomy surgery duration. Future studies will explore its potential applications to other surgery procedures and in improving surgeons' performance and training novices.

## 1. Introduction

There is a long-standing interest among medical professionals to evaluate surgical outcomes resulting from the advancement of surgery techniques. The traditional approach is to compare different procedures, such as open surgery versus endoscopic procedures, with regard to physical concepts and statistical analyses. Usually, the advantages and disadvantages of two surgical procedures can be compared in terms of duration of operation, recurrence rate, complications, postoperative pain, cost, and duration of hospital stay [1]. Such comparisons are also useful for teaching and training purposes or estimating the dexterous skill [2–4]. However, the skill level varies among surgeons with different levels of training and experience [5, 6]. Because operating equipment, techniques, and procedures have become increasingly complex over the past decades, discrete comparisons using traditional distribution charts of the parameters, such as surgery duration, may reveal tendencies but fail to discriminate between procedures at a satisfactory level of granularity. The tendency toward complexity adds difficulty in applying these comparative results for teaching and training purposes, and lack of granularity of data forces a surgeon to improve his/her specific surgical skills largely based on personal experience rather than scientific methods.

There are many possible methods to solve this issue. One practical way is dexterity analysis [7, 8]. In general, dexterity analysis is based on descriptive statistical analysis [9–13] and a language model of structural decomposition [14–16]. Dexterity analysis is different from the aforementioned types of general comparison in that it focuses narrowly on the specific tasks that the surgeon performs, rather than viewing comprehensively the general parameters mentioned above. Compared with other methods, dexterity analysis allows capture of the key features of the surgery and better presents the structure of the surgery. It also affords integration of the discrete data into a model for surgical training.

The present work differs from the existing dexterity analysis by aligning the tasks performed with the objectives and procedures of which they are a part and by connecting them with a simulation approach to estimate a distribution of surgery duration. For the purpose of simulating surgical procedures and improving the performance of surgeons, we propose to integrate dexterity analysis introduced above with a tool from engineering design—the design structure matrix [17, 18]. Design structure matrix (DSM) simulation can be used as a tool to integrate the result of dexterity analyses, such as hierarchical decomposition, with a probabilistic approach to evaluate the complete surgical procedure. The integrated DSM represents the important patterns of all activities in a dexterity language model, while the uncertainty of activity, such as rework [19, 20], corresponds to the dexterity descriptive data. This innovative application of DSM to surgery not only combines the two views of dexterity analysis but also provides a numerical simulation to predict the duration of a modified surgical procedure.

The present work takes laparoscopic cholecystectomy (LC), one of the most common surgical procedures in the US, as an example to illustrate the proposed innovative framework for surgery simulation. The LC surgical activities are integrated as the matrix inputs of the numerical DSM. The proposed framework for surgery simulation is validated by two standard protocols from surgical textbooks. An alternative LC surgical procedure is suggested by the *Atlas of Laparoscopic Surgery*, which may reduce surgery time. However, implementation of such modified procedure would incur time and cost for retraining surgeons. The current work enables estimation of the possible benefit in terms of shorter operation time that the suggested alternative surgical procedure may entail and helps the surgeon determine whether to adopt the modified procedure.

In light of the introduction above, the objectives of the present work are (a) to develop a modeling framework based on integration of dexterity analysis and DSM, which can be generally applicable to predict total durations of surgical procedures, and (b) to validate the model by comparing its results with the LC surgery protocol. Although dexterity analysis has been applied to the analysis of surgical procedures in previous studies, the model developed in this work represents an innovative approach that no previous study has ever reported. In particular, this is the first work where (a) DSM is applied to analyze surgical procedures and (b) dexterity analysis and DSM techniques are integrated and applied to the analysis of surgical procedures.

## 2. Method

The current numerical model was developed in two phases. Phase 1 involved the generation of hierarchical structures of dexterity analysis [7] in which corresponding data based on video samples were collected and analyzed. In Phase 2, a numerical DSM was developed to predict duration for different surgical procedures by generating the statistical distributions for comparison [17, 18].

This work involves secondary analysis of de-identified existing data and is eligible for exemption of review by the local institutional review board.

### 2.1. Model Development for Simulation of Surgery

**Figure 1:**
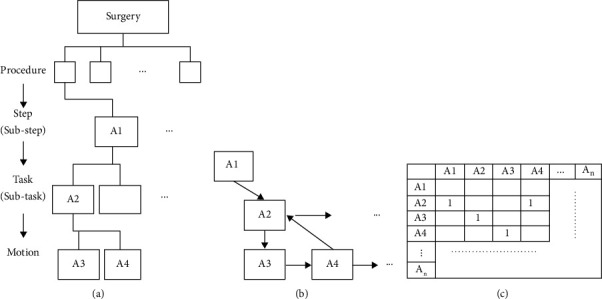
Integration of hierarchical dexterity analysis and DSM.

As stated as an objective above, a model based on integration of dexterity analysis and DSM is developed in this study to predict the total duration of a surgical procedure. Development of the model involves the following procedures: (1) decomposing the surgical procedure recorded by video into activities through dexterity analysis, (2) identifying the interactions among all the surgical activities and constructing a network, (3) building a DSM to show details of the interactions among all surgical activities, and (4) developing an algorithm to calculate the surgery duration based on the DSM.  Figure 1 illustrates the steps in integrating hierarchical dexterity analysis with the DSM using the video data: The surgical procedure is first decomposed into a number of activities through dexterity analysis in Figure 1(a). The interactions among all activities are identified in Figure 1(b), and a DSM showing interactions among activities is constructed in Figure 1(c).

#### 2.1.1. Dexterity Analysis

The video surgical data collected were compared with the surgical protocol. The surgical protocol defines the standard surgical activity for the purpose of characterization or categorization of surgical activity extracted from surgical videos. Protocol analysis refers to the characterization of surgical activities captured from video to match the definitions of the activities in a standard surgical protocol. The significant advantage of protocol analysis compared with other analyses is producing a systematic model to describe the relationships among the activities [21]. The current protocol analysis was based on the design concepts of function behavior structure [22], and categories were created to provide an understanding of the processes in the model and to aid analysis of the surgical activities. This method defines the standard for how a particular task should be executed set by experienced and seasoned instructors or experts in the discipline. The protocol analysis of the surgical activities recorded in the videos provides the required information for the surgery modeling. The general protocol considered the standard in the current field of LC can be found in the *Atlas of Minimally Invasive Surgery* (AMIS, [1]).

The goal of dexterity analysis is to decompose surgical procedures into modular and reusable activity segments, termed surgemes. Cao et al. [23] first defined several surgemes as common surgical tasks using a tool/tissue and tool/object vocabulary consisting of actions based on the interaction between the tool and the environment. In their work, different levels of activities were recorded through a data collection system, such as VCR, and then segmented using a computerized system for video annotating and coding to analyze these surgemes in terms of the average times taken to execute the surgemes, the number of component motions, and the number of attempts for each motion to achieve the goals of the surgery. MacKenzie et al. [24] further developed a hierarchical approach for assessment based on plans and the structure of goal-directed human behavior on videotaped laparoscopic Nissen fundoplications. This hierarchical decomposition approach was extended to analyzing complex systems that involve the surgeon and operating room team [14, 16, 25, 26]. Decomposition of surgical procedures by these analytical methods is important as it defines the surgemes required for DSM modeling. As illustrated in Figure 1(a), the first step of the current analytical method takes a hierarchical approach for dexterity analysis by decomposing the surgical procedure into a number of surgemes (activities).

Due to the small sample size and large variation, the current surgical video data for the decomposed activities are represented as triangular distributions using the maximum and minimum values identified. The triangular distribution requires only three data points for each activity: optimistic or the best case value (BCV), most likely value (MLV), and pessimistic or the worst case value (WCV). The triangular probability density function (PDF) is defined by BCV, MLV, and WCV of each activity.

#### 2.1.2. Design Structure Matrix

A DSM model was developed to delineate the relationships among the activities involved in the surgical procedure; this model was then used for simulation. The concept of DSM has been widely applied to various categories of engineering design, engineering management, management/organization science, and systems engineering [18], and even communication in healthcare [27]. A DSM displays the relationships between the activities of a process in a compact, visual, and analytically advantageous format [17]. It is a square matrix with identical row and column labels. For each activity, its corresponding column(s) represent(s) the input(s) of the activity and the row(s) represent(s) its output(s) [19]. The number “1” in an off-diagonal cell indicates an input and output link between a pair of activities. An empty cell (or “0”) indicates no relationship between the activities. The diagonal cells are blank to preclude self-linking of each activity. More precise estimation of interactions between activities can be represented if different numbers (2, 3,…, etc.) are employed.

#### 2.1.3. Calculation of Surgery Duration

*(1) Definitions of Input Parameters*. During the surgery, some activities may need to be repeated before the subsequent activities may proceed. The total time duration required to complete the surgery therefore depends on three parameters—rework probability, rework impact, and improvement curve—of each activity included in the surgical procedure.

*Rework probability* (RP) is the probability that an activity needs to be repeated in order to correct or to repair a defective or failed trial of the activity. For an activity that produces an output based on the inputs of preceding (upstream) activities, rework (repetition) of the activity is required if the (upstream) input to the activity changes, and such change in the input causes the activity itself not to be able to produce a satisfactory output and thus needing to repeat. Theoretically, each input to an activity has a probability of change defined as volatility [28, 29] and a probability of a change in the input causing rework of the activity defined as sensitivity [28, 30]. These probabilities are multiplied to determine the rework probability (RP) for the activity caused by the change in the input:

(1) $$\begin{array}{c} P_{ir}=\;P_{i}\times P_{r}, \end{array}$$

where

 
*P*_*ir*_ = the probability that a change occurs to an input activity “*I*” of a certain activity “*A*” and resulting in a rework of “*A*”
 
*P*_*i*_ = the probability of change in the input activity “*I*”; i.e., “volatility”
 
*P*_*r*_ = the probability that the activity “*A*” needs rework due to this change in the input activity “*I*”; i.e., “sensitivity”

In this study, using the surgical video data, *P*_*i*_ is estimated:

(2) $$\begin{array}{c} P_{i}=\frac{N_{oc}}{N_{o}}, \end{array}$$

where

 
*N*_*o*_ = the total number of times that a certain input activity “*I*” of a certain activity “*A*” is observed in the surgical procedure
 
*N*_*oc*_ = among *N*_*o*_, the number of times that the input activity “*I*” is performed in an unusual way (i.e., involving certain change/deviation) compared with the standard procedure defined by the protocol

*P*_*r*_ can be calculated by the following equation:

(3) $$\begin{array}{c} P_{r}=\;\frac{N_{ir}}{N_{ic}}, \end{array}$$

where

 
*N*_*ic*_ = the total number of times that the input activity “*I*” is performed in an unusual way (i.e., involving certain change/deviation) compared with the standard procedure defined by the protocol
 
*N*_*ir*_ = among *N*_*ic*_, the number of times causing rework of the subsequent activity “*A*”

Note that *N*_*ic*_ − *N*_*ir*_ = the number of times that the change in “*I*” does not cause rework of “*A*.” Different types of changes in an input activity may have different probabilities of causing rework of the subsequent activity.

*Rework impact* (RI) is the impact of rework on an activity, i.e., the portion of a particular activity to be reworked:

(4) $$\begin{array}{c} \text{RI}=\;\frac{W_{r}}{W_{\text{total}}}=\frac{D_{r,nic}}{D_{o}}, \end{array}$$

where

 
*W*_*r*_ = the amount of work that needs to be repeated in the activity, i.e., the amount of rework
 
*W*_*total*_ = the total amount of work in the activity
 
*D*_*r*,*nic*_ = duration required to complete the rework in the activity without considering the improvement-curve effect
 
*D*_*o*_ = duration for the first execution of the activity before any rework, i.e., the original duration

While some changes causing rework can possibly be absorbed by a robust activity with little impact [31], the consequences of other changes may be more severe. A typical approach to estimating RI is through interviewing experts to solicit feedback based on their professional skills and experience [29, 32]. In the present study, senior surgeons were interviewed for such purpose. The RI for each activity is evaluated for various situations including extreme cases (e.g., bleeding or complication) and different operation areas (e.g., left or right side of gallbladder). A range of possible RI values was generated, and the average was calculated for the simulation.

An *improvement curve* (IC or *learning curve*) quantifies the decrease of time required to perform the activity with experience and is defined as

(5) $$\begin{array}{c} \text{IC}=\;\frac{D_{r,ic}}{D_{r,nic}}, \end{array}$$

where *D*_*r*,*ic*_ is the reduced duration required to complete the rework of the activity due to the improvement-curve effect.

Various models for learning curves in surgical practice have been proposed [33]. In general, IC is related to RI because it may take less time to rework an activity than the first time. Thus, IC needs to be modeled for each activity as a step function, where an activity initially takes 100% of its duration to accomplish, while the subsequent executions of the activity take less than the original duration [28]. In principle, the learning curve could be estimated statistically using data collected from a surgeon's training program. If the surgeons are experienced and have performed the surgery numerous times after rigorous professional training, and that their execution of the surgical activities is considered to have little room to improve, IC is taken as a constant at 100% (no improvement).

Based on the above definitions, the duration for rework of a particular activity is calculated as

(6) $$\begin{array}{c} D_{r,ic}=D_{0}\times \;\frac{D_{r,nic}}{D_{o}}\times \;\frac{D_{r,ic}}{D_{r,nic}}=D_{0}\times \text{ RI}\times \text{ IC}. \end{array}$$

*(2) Simulation Algorithm*. The current simulation algorithm is based on a series of works in the fields of systems engineering and management by Browning and coauthors [17, 28, 34, 35]. This is the first application of such works to surgery.

The simulation begins with input of a random duration data based on a triangular probability distribution established by data from the surgery videos for each surgical activity. The simulated surgery then proceeds by executing as many activities as possible following the sequence established in the DSM, until encountering an activity that depends on certain upstream activity/activities that have not yet been executed, or no further downstream activity exists (i.e., end of the surgical procedure). This group of executed activities constitutes an active set that appears as a submatrix in the DSM. The total duration of the activities in this active set is then calculated by adding the durations of all activities in this set.

For a rework loop involving two activities *A* and *B*, for example, a random probability between 0 and 1 is first generated (using RND function in the Visual Basic macro of the simulation program) for the simulation value of rework probability of *B* due to a change in its input *A*, *SP*_*ir*,*A* −* B*_. Rework of *B* continues if *SP*_*ir*,*A*−*B*_ > *P*_*ir*,*A*−*B*_, until *SP*_*ir*,*A*−*B*_ ≤ *P*_*ir*,*A*−*B*_, where *P*_*ir*,*A*−*B*_ is the actual rework probability of *B* due to a change in *A* based on observation (of surgery video), as specified in the DSM. The total duration of the rework of the activity *B* due to a change in its input *A* in this rework loop is then calculated as follows:

(7) $$\begin{array}{c} D_{ra}=D_{0}\times \text{RI}\times \sum_{i=1}^{nr}{\left(\text{IC}\right)}_{i}, \end{array}$$

where

 
*D*_*ra*_ = duration required to complete activity (*B*) in a rework loop
 
*nr* = the number of rework times of activity (*B*) due to a change in its input (*A*), determined by the criterion *SP*_*ir*,*A*−*B*_ ≤ *P*_*ir*,*A*−*B*_

The same method applies to calculating the total duration of the rework of the activity *A* due to a change in its input *B* in this rework loop involving *A* and *B*. The same method is also applied to a rework loop involving more than two activities. For example, for a rework loop involving three activities *A*, *B*, and *C*, this method is first applied to rework of activity *B* due to a change of input activity *A*, then rework of activity *C* due to a change of input activity *B*, and finally rework of activity *A* due to a change of input activity *C*. The total time duration for a rework loop is

(8) $$\begin{array}{c} D_{rl}=\sum_{i=1}^{nal}{\left(D_{ra}\right)}_{i}, \end{array}$$

where *nal* is the number of activities involved in the rework loop.

For a surgical procedure involving multiple rework loops, the total simulation duration for the entire surgical procedure is then calculated as follows:

(9) $$\begin{array}{c} \text{Total duration}=\left(\text{Duration of the first trial}\right)+\left(\text{Total rework duration}\right),\\ D_{\text{total}}=\sum_{i=1}^{na}{\left(D_{o}\right)}_{i}+\sum_{i=1}^{nl}{\left(D_{rl}\right)}_{i}, \end{array}$$

where

 
*na* = the total number of activities in the surgical procedure
 
*nl* = the total number of rework loops in the surgical procedure

**Figure 2:**
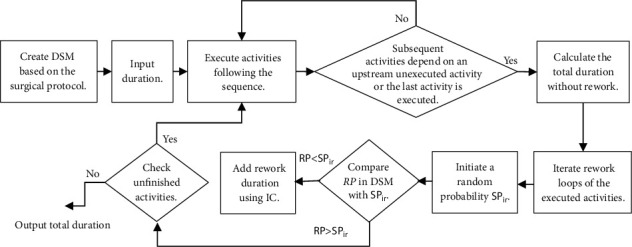
Flowchart of simulation algorithm.

DSM Program 2.0 Excel Macros for Partitioning and Simulation, available online (https://www.dsmweb.org), was modified according to the algorithm illustrated in Figure 2.

**Figure 3:**
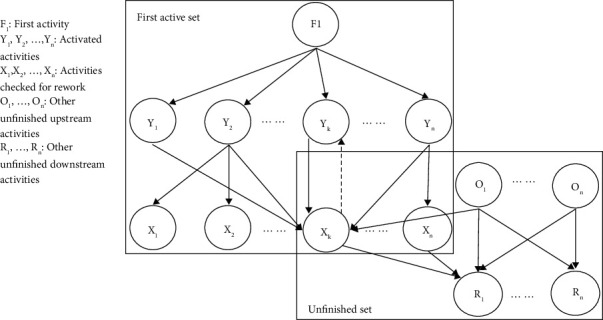
Schematic of simulation algorithm.

Figure 3 shows an example how the algorithm works for different levels of activities in a hierarchical decomposition. All the downstream activities *X*_*s*_ and *Y*_*s*_ related to the first activity *F*_1_ are identified and executed. Activities *Y*_*s*_ are then checked for dependence on any downstream activity *X*_1_ through *X*_*n*._ Probabilistic rework is the key to identify all the upstream activities that are dependent on the downstream activities that create rework loops. The relationship is illustrated by the broken arrow line between *X*_*k*_ and *Y*_*k*_ in Figure 3. To simulate the rework loop, the rework probabilities of the corresponding activity, the superdiagonal cells and subdiagonal cells in each column of the DSM are used to estimate the most probable number of rework times for the loop. In individual rework iteration, the rework duration of the activities in the loop is calculated using the input duration, rework impact, and learning curve. After the entire active set is simulated including rework loops, the first active set is ignored and the program will reexamine the new unfinished highest rank order activities *O*_*s*_ (*O*_1_,…,.*O*_*n*_) and unfinished downstream activities *R*_*s*_ (*R*_1_,…, *R*_*n*_). By running the simulation a number of times until no downstream activities remain, the total surgery duration is calculated by summing up all the durations of the sets.

*(3) Risk Factor in Simulation Algorithm*. Risk factors are the major parameters accounting for various unexpected simulation results. In a simulation, outliers of the simulation results represent significant consequences. The input parameters causing these outliers are the risk factors. The main simulation inputs in the present model include duration estimations (BCV, MLV, and WCV), *P*_*ir*_, RI, and IC. BCV, MLV, and WCV, according to the definition, are directly proportional to the duration output. IC is a constant in our case. Thus, only *P*_*ir*_ and RI need to be considered risk factors.

Browning and Eppinger [28] proposed the risk factor *R* for different consequences as a function of both the corresponding rework probability and rework impact:

(10) $$\begin{array}{c} R=P_{ir}\times RI, \end{array}$$

where *R* is the risk factor. The cumulative impact of *R* in the rework propagation is calculated by a step function [17]. The *n*-step risk of rework propagation for activity *A*_*ij*_ in row *i* and column *j* of an *m* × *m* DSM is estimated by the power function *R*^*n*^. Therefore, the maximum cumulative risk of rework propagation, *CR*, is

(11) $$\begin{array}{c} CR_{ij}=\sum_{n=1}^{\infty }R_{ij}^{n}={\left(1-R\right)}^{-1}-1,\;i=1,2,\ldots ,m;j=1,2,\ldots ,m;i\neq j. \end{array}$$

For an *m* × *m* DSM, let *f*_*j*_=∑_*i*=1_^*m*^*CR*_*ij*_,  *i*=1,2,…, *m*, and *e*_*i*_=∑_*j*=1_^*m*^*CR*_*ij*_,  *j*=1,2,…, *m*, where *f*_*j*_ is the total maximum risk transferred from activity *A*_*ij*_ to other activities and *e*_*i*_ is the total maximum risk that activity *A*_*ij*_ receives from the other activities. To measure the role that *CR*_*ij*_ of *A*_*ij*_ plays in the rework propagation caused by both output and input, the total risk *TR*_*ij*_ is calculated as follows:

(12) $$\begin{array}{c} TR_{ij}=\sum_{i=1}^{m}\sum_{j=1}^{m}CR_{ij},\;i=1,2,\ldots ,m;j=1,2,\ldots ,m;i\neq j. \end{array}$$

## 3. Results

### 3.1. Validation of Simulation Model

#### 3.1.1. Model Validation by Standard Laparoscopic Cholecystectomy Surgical Protocol

**Figure 4:**
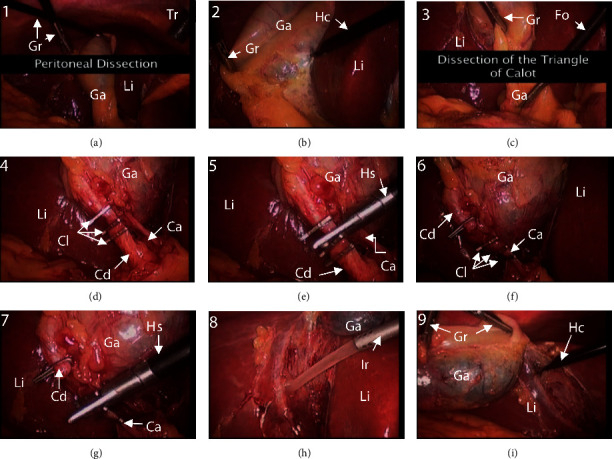
Surgical activities in AMIS video for laparoscopic cholecystectomy protocol: (a) peritoneal dissection, left side (activity *A*1); (b) peritoneal dissection, right side (activity *A*2); (c) dissection of the triangle of Calot (activity *A*3); (d) dissection and clipping the cystic duct (activity *A*4); (e) cutting the cystic duct (activity *A*5); (f) dissection and clipping the cystic artery (activity *A*6); (g) cutting the cystic artery (activity *A*7); (h) cleaning bile and/or blood (activity *A*8); and (i) separation of the gallbladder from liver (activity *A*9). Ca: cystic artery; Cd: cystic duct; Cl: clip; Fo: forceps; Ga: gallbladder; Gr: grasper; Hc: hook cautery; Hs: hook scissors; Ir: irrigator; Li: liver; Tr: trocar.

The present simulation model was first validated by comparing the duration results of its simulated surgery with that of the LC video in the *Atlas of Minimally Invasive Surgery* (AMIS, [1]). The recording and editing of the video were based on one chapter of the standard surgical protocol by Frantzides et al. [36]. This protocol was first chosen to validate the proposed model because it is short and simple and also considered a standard for the LC procedure. The video clearly presents the major activities involved in the surgery, with snapshots of the key activities shown in Figure 4. Detailed description of all activities can be found in Chapter 20 of AMIS [1].

**Figure 5:**
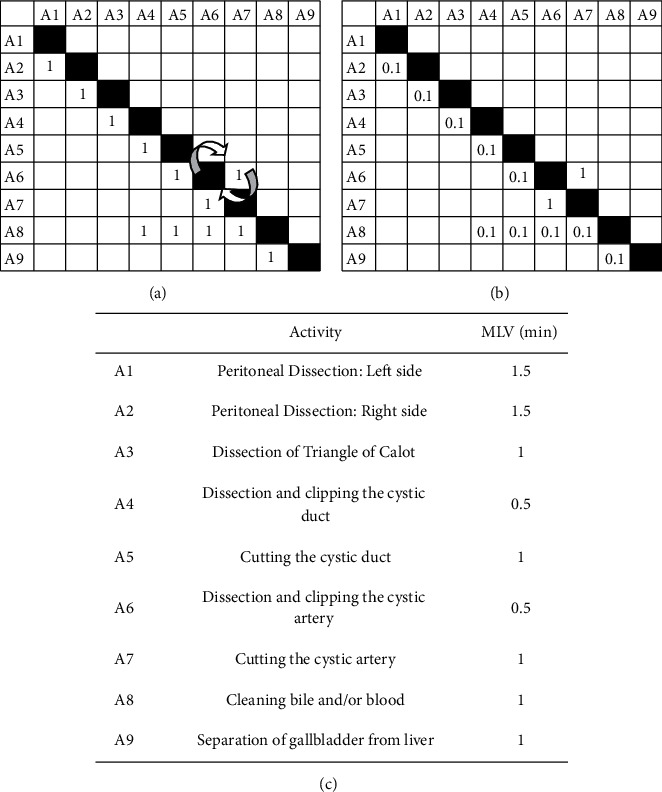
DSM, rework probability, rework impact, and activity duration data for the AMIS protocol. (a) DSM. (b) Rework probabilities and rework impacts for all activities. (c) Most likely values for activity durations.

*(1) DSM of AMIS Protocol.* Nine activities (*A*1–*A*9) are identified in the AMIS video (see Figure 4), with the input DSM shown in Figure 5. There are few deviations from the normal activities and few reworks in this standard procedure video, which may have been edited for training purpose. A single rework loop in Figure 5(a) is identified as associated with activity *A*6 (dissection and clipping the cystic artery) and activity *A*7 (cutting the cystic artery). These two activities are reworked due to bleeding when the cystic duct and artery are dissected or cut. As a result of the bleeding, the rework probabilities of this loop are very high (taken as 1 because there is only one sample). Although no other rework is observed in this training video, the RPs of the other activities are all taken as 0.1 as a realistic estimation. Other than this loop, the interactions between *A*4–*A*7 and *A*8 (marked by cells below the diagonals in Figures 5(a) and 5(b)) represent the additional cleaning (*A*8) required due to serious leakage of bile from the cystic duct and/or bleeding from the cystic artery caused by dissection and cutting activities (*A*4–*A*7). Coincidentally, RIs are the same as RPs in this case.

*(2) Result of Simulation Model Compared with AMIS Protocol.* Based on the MLV data for the durations of all activities (Figure 5(c)), the total duration of the surgical procedure is calculated as 10 minutes, which is in excellent agreement with that of the AMIS surgery protocol of approximately 9 minutes and 45 seconds (2.5% error). The primary reason causing the error may be related to estimations of MLVs. Based on the video, it is very difficult to estimate the activity durations with better than 0.1-minute accuracy.

#### 3.1.2. Model Validation by Pediatric Laparoscopic Cholecystectomy Protocol

The present simulation model was also validated by comparing its prediction result with that of the LC video in the *Atlas of Pediatric Laparoscopy and Thoracoscopy* (APLT, [37]). The video was short and well edited based on Chapter 22 of the standard surgical protocol by Holcomb et al. [37].

**Figure 6:**
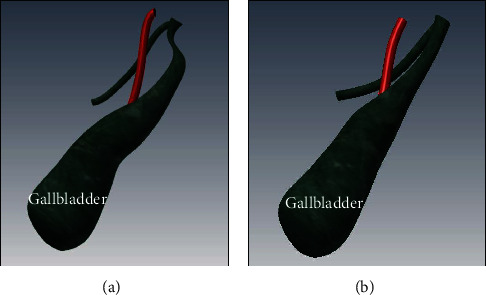
Difference between (a) pediatric laparoscopic cholecystectomy protocol (APLT) and (b) standard laparoscopic cholecystectomy surgical protocol (AMIS).

Most of the activities in this APLT  protocol are similar to AMIS, with the only difference being in the activities related to dissection of the triangle of Calot suggested by Holcomb et al. [37] particularly for pediatric LC surgery. The triangle of Calot, also known as the hepatobiliary triangle, or cystohepatic triangle, is an anatomic space bordered by the hepatic duct medially, the cystic duct laterally, and the cystic artery superiorly. The APLT procedure features grasping the infundibulum of the gallbladder (distal to the cystic duct) using the grasping forceps and retracting inferiorly and laterally. Such a treatment creates a larger angle (90°) between the cystic duct and the common bile duct (Figure 6(a)) than the AMIS procedure (Figure 6(b)). It is especially useful in pediatric application because it can avoid accidentally cutting the common bile duct while attempting to cut the cystic duct. Although it entails more activities in the DSM input, thus increasing the matrix size, the APLT procedure has certain advantages such as no loop connected to any other activity in the DSM compared with the AMIS protocol.

**Figure 7:**
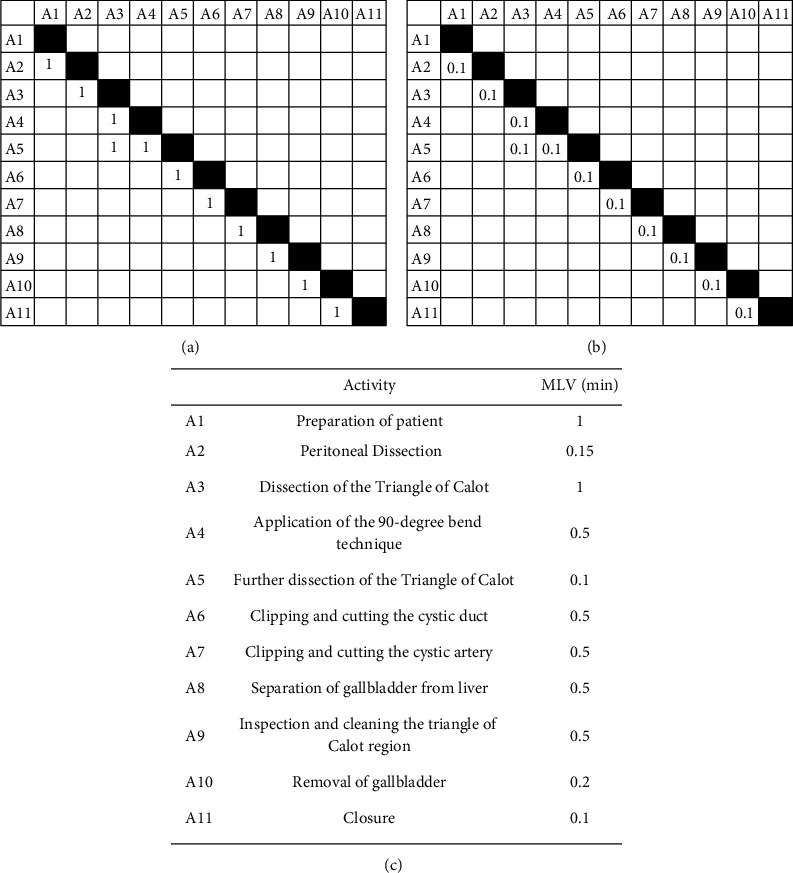
DSM, rework probability, rework impact, and activity duration data for the APLT protocol. (a) DSM. (b) Rework probabilities and rework impacts for all activities. (c) Most likely values for activity durations.

*(1) DSM of APLT Protocol.* The video for APLT has been edited with significantly shortened durations of the activities just to demonstrate the surgical procedures. Nevertheless, our model can be applied to estimate the total duration based on the edited video. Eleven activities were identified in this video, with the DSM shown in Figure 7(a) and no loop identified. The RPs of the activities are all zero (no reworks), while the RPs and RIs are taken as 0.1 for a realistic estimation, similar to the analysis of AMIS above. The direct relationship between rework probability and rework impact is shown in Figure 7(b).

*(2) Result of Simulation Model Compared with APLT Protocol.* The total duration of the APLT surgery protocol is 5 minutes and 30 seconds, while the current simulation model based on data in Figure 7(c) yields 5.7 minutes, which is within 4% error compared with the APLT protocol. The error is larger compared with validation with AMIS, most likely due to the errors associated with estimating the durations of a larger number of activities involved in the procedure.

## 4. Discussion

The present simulation framework provides a tool for analyzing surgical procedure and predicting surgery duration. It integrates dexterity analysis and DSM to provide a clear and simple insight of the complex structure inherent in a surgical procedure. Validation of the present framework by comparing with two standard surgery protocols represents a progress toward better prediction of the total surgery duration for not only laparoscopic cholecystectomy but also possibly other types of surgery that can be simulated. Prediction of surgery duration is important as prolonged surgery time may be associated with anesthesia time, blood loss, tissue-air exposure time, risk of infection, surgical insult, surgeon's performance, among others.

This work is limited in terms of validation. In addition to the validation by the standard LC videos developed for teaching purpose reported in this work, the present simulation model can be further validated by comparing its results with LC videos documenting the entire surgical procedure performed by different surgeons on different patients. The surgery duration predicted by the present model can be compared with the duration of the video samples. One way to generate more accurate simulation results is to collect the video samples of only one surgeon operating on multiple patients. Detailed clinical information such as gender, body mass index, previous surgery, inflammation history, and sonographic diagnosis can also be integrated into an advanced version of the model. However, these additional parameters may depend more on empirical knowledge and subjective evaluation. It is easy to determine statistical parameters associated with age, gender, and body mass index, but parameters associated with previous surgery or sonographic diagnosis may have false positive or false negative impact on the estimation of the total surgery duration. This problem may be solved by modeling specific surgery procedures with highly standardized algorithms developed particularly for a certain type of surgery.

The present model demonstrates a potential application in improving surgeon's performance by eliminating factors causing variation of the operation time, estimating change of operation time by modifying a part of the surgical procedure, analyzing advantages and disadvantages of such modification, and comparing with standard protocol. The surgery procedure can be improved by decoupling activities identified in the DSM in order to reduce complexity of the operation procedure, and our model can further predict how much total surgery duration is reduced by such decoupling. In fact, the present model can evaluate any alternative techniques that may reduce the total surgery duration.

Another possible application of the current model is to help train novices and improve their dexterity of the surgical procedures. In the current framework, input parameters such as rework probability and rework impact are employed to predict the total surgery duration, and these parameters can also be employed for training purposes. A novice's performance can be evaluated by examining these data collected from the dexterity training as the model's input parameters. In particular, the present framework can be an effective tool to train novices by reducing large differences between BCV and WCV and lowering high rework probabilities associated with their surgical activities identified using the present tool.

## 5. Conclusion

Tools for improving surgical outcomes, such as reducing the duration of common surgical procedures, are highly sought after by medical professionals. The current study contributes to this goal by providing a simulation framework that can predict the duration of a surgical procedure based on analysis of the activities performed during the procedure, rather than statistical analysis of a procedure as a whole. The framework presented combines dexterity analysis of surgery videos, modeling of the procedure using DSM, and calculation of the surgery duration. The current study develops and validates a numerical DSM simulation model of the entire laparoscopic cholecystectomy surgery procedure, including couplings (loops) and reworks identified. Further study will test this model as a tool to investigate the advantages and disadvantages of modified and alternative surgical activities, to test surgical procedure redesign, and to compare different versions of surgical protocol through simulation.

## Data Availability

The data sets generated during and/or analyzed during the current study are available from the corresponding author on reasonable request.

## Nomenclature

*D*: Time duration
IC: Improvement curve (learning curve)
*N*: Number
*P*: Probability
RI: Rework impact
*SP*: Simulation probability
W: Work

### Subscripts

*a*: Activity
*c*: Change
*i*: Input
*ic*: Improvement curve
*l*: Loop
*n*: Number
*nal*: Number of activities involved in the rework loop
*nic*: Without improvement-curve effect
*nr*: Number of times of rework
*o*: Observed
*r*: Rework.
